# Dual-functional application of Ca_2_Ta_2_O_7_:Bi^3+^/Eu^3+^ phosphors in multicolor tunable optical thermometry and WLED

**DOI:** 10.1007/s12200-024-00134-2

**Published:** 2024-09-04

**Authors:** Jingjing Ru, Bing Zhao, Fan Zeng, Feiyun Guo, Jinhua Liu, Jianzhong Chen

**Affiliations:** 1 College of New Energy and Materials, Fujian Province University Key Laboratory of Green Energy and Environment Catalysis, Ningde Normal University, Ningde, 352100 China; 2 College of Mechanical and Electrical Engineering, Ningde Normal University, Ningde, 352100 China; 3 School of Environment and Resources, School of Carbon Neutral and Modern Industry, Fujian Normal University, Fuzhou, 350007 China; 4 College of Chemistry, Fuzhou University, Fuzhou, 350108 China; 5 School of Pharmacy and Medical Technology, Key Laboratory of Pharmaceutical Analysis and Laboratory Medicine of Fujian Province, Putian University, Putian, 351100 China

**Keywords:** Phosphor, Energy transfer, Zero-thermal-quenching, Optical thermometry, WLEDs

## Abstract

A series of Bi^3+^/Eu^3+^ co-doped Ca_2_Ta_2_O_7_ (CTO:Bi^3+^/Eu^3+^) phosphors were prepared by high-temperature solid-state method for dual-emission center optical thermometers and white light-emitting diode (WLED) device. By modulating the doping ratio of Bi^3+^/Eu^3+^ and utilizing the energy transfer from Bi^3+^ to Eu^3+^, the tunable color emission ranging from green to reddish-orange was realized. The designed CTO:0.04Bi^3+^/Eu^3+^ optical thermometers exhibit significant thermochromism, superior stability, and repeatability, with maximum sensitivities of *S*_a_ = 0.055 K^−1^ (at 510 K) and *S*_r_ = 1.298% K^−1^ (at 480 K) within the temperature range of 300−510 K, owing to the different thermal quenching behaviors between Bi^3+^ and Eu^3+^ ions. These features indicate the potential application prospects of the prepared samples in visualized thermometer or high-temperature safety marking. Furthermore, leveraging the excellent zero-thermal-quenching performance, outstanding acid/alkali resistance, and color stability of CTO:0.04Bi^3+^/0.16Eu^3+^ phosphor, a WLED device with a high *R*_a_ value of 95.3 has been realized through its combination with commercially available blue and green phosphors, thereby demonstrating the potential application of CTO:0.04Bi^3+^/0.16Eu^3+^ in near-UV pumped WLED devices.

**Graphical abstract:**

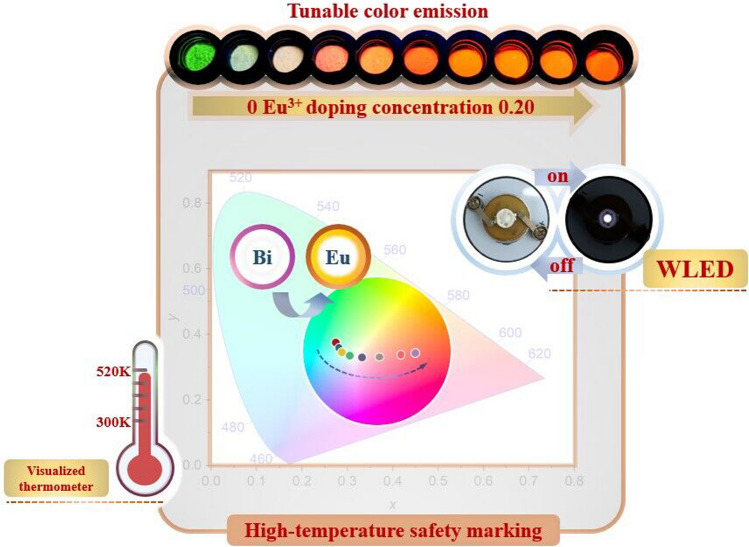

**Supplementary Information:**

The online version contains supplementary material available at 10.1007/s12200-024-00134-2.

## Introduction

Currently, white light-emitting diodes (WLEDs), particularly phosphor-converted WLEDs (pc-WLEDs) [1–5], have been vastly deemed the most remarkable new generation solid-state lighting sources due to their superior light emission efficiency, energy-saving, and environmental properties, which have penetrated into various fields, such as indoor and outdoor lighting, biological technologies, and plant lighting [6–9]. However, a significant challenge for pc-LEDs is the occurrence of thermal quenching (TQ) effects in most reported phosphor materials at elevated temperatures, which severely limits their widespread use in practical real-world environments [10–14]. Recently, several strategies including phase transition regulation, lattice defects design, structure tailoring, energy transfer (ET) and coating methods have been applied to develop phosphors with zero-thermal-quenching performance [15–19]. Leng et al. reported a blue phosphor Na_3_KMg_7_(PO_4_)_6_:Eu^2+^ with zero-thermal-quenching performance for the first time, which was attributed to its large band gap structure [20]. Wu et al. demonstrated zero-thermal-quenching behavior in a blue phosphor K_m-0.4_Al_11_O_17 + δ_:0.2Eu^2+^ within the temperature range of 423 K, which was attributed to the rigid aluminate structure and the introduction of trap energy levels [21]. Wang et al. conducted research on a novel red phosphor LaSc_3_(BO_3_)_4_:Eu^3+^, which exhibited high-temperature zero-thermal-quenching behavior under thermal disturbances. This was attributed to a high rigid crystal structure and a weak concentration quenching effect [22]. The LiAl_5_O_8_: Eu^2+^, Mn^2+^ blue-green emitting phosphor studied by Liu et al. exhibited zero-thermal-quenching at 423 K, attributed to energy transfer from Eu^2+^ to Mn^2+^ [23]. However, there is still limited research and information available on such materials. Consequently, enhancing the TQ performance of phosphors remains a significant challenge in the advancement of pc-WLEDs.

Temperature, as a crucial physical parameter in assessing hot and cold conditions, is significantly intertwined with human production and daily life [24–26]. The conventional method for measuring temperature relies on contact measurement, necessitating direct contact between the sensor and the object being measured to facilitate sufficient heat exchange. However, this approach proves inadequate when addressing measurement requirements in challenging and intricate environments, such as high temperature, corrosion, electromagnetic interference, and micron-scale measurement [27–29]. Moreover, this approach also exhibits drawbacks including low sensitivity, prolonged response time, and high costs. In contrast, non-contact optical thermometers present a promising solution to address the afore-mentioned challenges and have become an area of intense research.

Temperature detection typically involves monitoring specific photoluminescence parameters that are sensitive to changes in temperature, such as fluorescence intensity ratio (FIR), fluorescence intensity, attenuation lifetime, color coordinates, and emission band shift, which could allow for accurate and reliable measurement of temperature variations in a given system [30–35]. Among which, FIR thermometers have gained significant attention as a non-contact temperature measurement technique, owing to their extensive detection temperature range, notable accuracy, and high resolution [36]. Traditionally, FIR thermometers are based on the fluorescence intensity ratio of two thermally coupled energy levels (TCELs) of a single rare earth (RE) ion center [37–39]. However, these ones exhibit low relative sensitivity (*S*_r_) at higher temperatures due to the fixed band gap (200−2000 cm^−1^) between the TCELs. To overcome this dilemma, a strategy of designing dual-emitting centers with different thermal responses has been proposed. In recent years, FIR thermometers based on co-doped phosphors with RE/RE or RE/NRE (non-rare earth) ions pairs have been extensively investigated. These pairs may be Eu^3+^/Sm^3+^, [40] Bi^3+^/Sm^3+^ [41], Mn^4+^/Eu^3+^ [42], Eu^2+^/Eu^3+^ [43], Pr^3+^/Tb^3+^ [44], Yb^3+^/Mn^2+^ [45], Bi^3+^/Eu^3+^ [46–48], and so on. Luo et al. investigated the utilization of YNbO_4_:Bi^3+^, Sm^3+^ phosphors, employing both color coordinates and fluorescence intensity ratio to develop a dual-mode optical temperature design [41]. In a separate study, Li et al. examined Ca_2_GdSbO_6_:Mn^4+^/Eu^3+^ and Ca_2_GdSbO_6_:Mn^4+^/Sm^3+^ phosphors as non-contact optical thermometers [42]. These phosphors exhibited the *S*_r_ of 1.38% K^−1^ (at 420 K) and 1.55% K^−1^ (at 430 K), respectively, demonstrating their potential application for accurate temperature measurement at high temperatures. In another instance, Wu et al. synthesized LuNbO_4_:Pr^3+^, Tb^3+^ phosphors, which possess the capability to serve as self-calibrating optical thermometers [44]. The exceptional sensitivity of these phosphors can be attributed to the disparity in thermal activation energy between Pr^3+^ and Tb^3+^ ions, rendering them as promising candidates for precise temperature measurements. In essence, these studies make a substantial contribution to the evolution of optical temperature measurement techniques, presenting a plethora of options tailored for diverse applications while demonstrating the inherent potential of various phosphor materials within this realm.

Bi^3+^ ions, with a 6s^2^ configuration, exhibit exceptional sensitivity to the crystal field of the surrounding matrix [49, 50]. By modulating the crystal field environment, the emission color of Bi^3+^ ions can be effectively regulated, ranging from ultraviolet to red light. In contrast, Eu^3+^ ions with a 4f [6] configuration are less prone to temperature-induced effects due to the shielding effect of their 5s^2^5p [6] electrons on the 4f [6] electrons [29, 51]. Consequently, the development of a suitable matrix doped with both Bi^3+^ and Eu^3+^ as dual emission centers, holds great promise as an ideal optical temperature sensing material, enabling simultaneous distinct emission bands and tunable emission colors, with significant potential for diverse applications.

Pyrochlore structure-type A_2_B_2_O_7_ has been widely used in various applications, such as thermal barrier coatings, fluorescent materials, high-temperature fuel cells, and oxidation catalysts, owing to its intricate composition, structure, and remarkable optical, electrical, and magnetic properties [52, 53]. The wide-bandgap ternary metal oxide Ca_2_Sb_2_O_7_, renowned for its compact structure, has emerged as a prominent phosphor matrix material in recent years due to its exceptional photoluminescent properties [54, 55]. Particularly, the Bi^3+^/Eu^3+^ co-doped Ca_2_Sb_2_O_7_ phosphor has garnered extensive interest for its versatile applications in non-contact optical temperature sensing and thermochromism [56]. Ca_2_Ta_2_O_7_, characterized by its outstanding physical and chemical stability, has been effectively utilized as a photocatalytic material in the realms of photocatalytic hydrogen generation and photodegradation [57]. However, its utilization in WLEDs remains relatively uncommon. This is especially true for Bi^3+^/Eu^3+^ co-doped Ca_2_Ta_2_O_7_ materials, which have yet to be extensively explored in the field of optical thermometry. Leveraging the FIR temperature measurement strategy, a set of Ca_2(1−*x−y*)_Ta_2_O_7_:*x*Bi^3+^/*y*Eu^3+^ (abbreviated as CTO:*x*Bi^3+^/*y*Eu^3+^) optical thermometers with two correlated signals simultaneously were successfully designed. Meanwhile, the potential application in WLED device was also explored.

## Experimental

CTO:*x*Bi^3+^ (*x* = 0, 0.005, 0.01, 0.02, 0.03, 0.04, and 0.05) and CTO:0.04Bi^3+^/*y*Eu^3+^ (*y* = 0.01−0.20) samples were synthesized using a high-temperature solid-state reaction method. To obtain the desired product, precise quantities of raw materials, namely CaCO_3_ (99.99%), Bi_2_O_3_ (99.9%), Eu_2_O_3_ (99.99%), and Ta_2_O_5_ (99.99%) were meticulously weighed according to the stoichiometric ratio. Taking CTO:0.04Bi^3+^/0.01Eu^3+^ as an example, 3.33 mmol of CaCO_3_ (0.3337 g), 0.068 mmol of Bi_2_O_3_ (0.0318 g), 0.017 mmol of Eu_2_O_3_ (0.0060 g), and 1.75 mmol of Ta_2_O_5_ (0.7733 g) were added into an agate mortar. Vigorous grinding was performed for 0.5 h. Subsequently, the ground powder was transferred to an alumina crucible and subjected to sintering at 1723 K for a duration of 15 h. After cooling, the material was further processed by grinding into a powder for subsequent analyses. The detailed preparation process of WLED device and characterizations could be found in the supporting information.

## Results and discussion

### Microstructure analysis

**Fig. 1:**
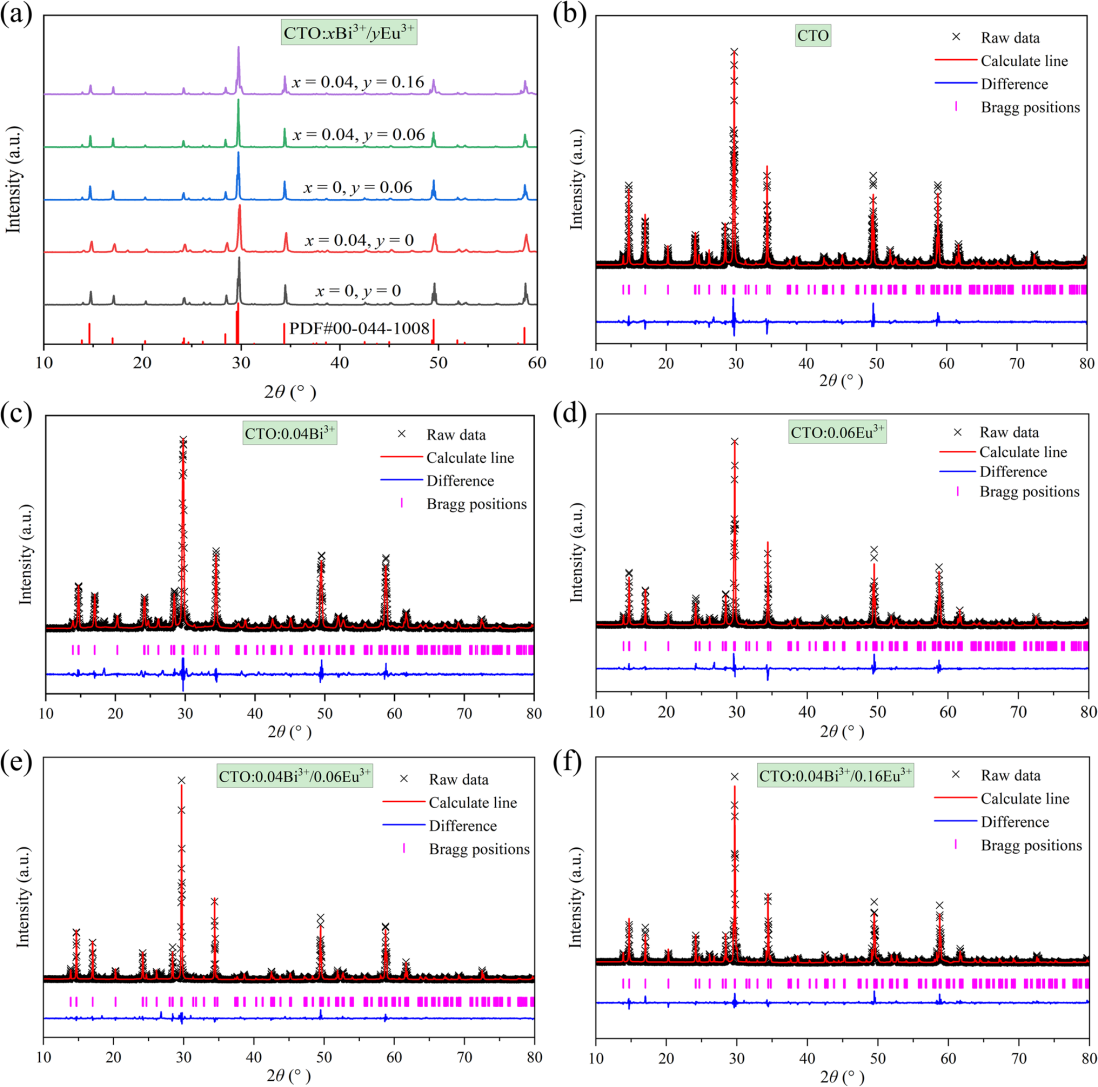
**a** XRD patterns of CTO:*x*Bi^3+^/*y*Eu^3+^. Rietveld refinements for **b** CTO, **c** CTO:0.04Bi^3+^, **d** CTO:0.06Eu^3+^, **e** CTO:0.04Bi^3+^/0.06Eu^3+^, and **f** CTO:0.04Bi^3+^/0.16Eu^3+^

The XRD patterns of CTO, CTO:0.04Bi^3+^, CTO:0.06Eu^3+^, CTO:0.04Bi^3+^/0.06Eu^3+^ and CTO:0.04Bi^3+^/0.16Eu^3+^ samples were illustrated in Fig. 1a, and the ones of the prepared CTO:*x*Bi^3+^ (*x* = 0.005, 0.01, 0.02, 0.03, and 0.05) and CTO:0.04Bi^3+^/*y*Eu^3+^ (*y* = 0.01, 0.02, 0.04, 0.08, 0.12, and 0.20) were shown in Fig. S1. Except for CTO:0.04Bi^3+^/0.20Eu^3+^ (*y* = 0.20) sample, all primary diffraction peaks, devoid of impure phases, match well with the standard card of hexagonal phase Ca_2_Ta_2_O_7_ (PDF#00-044-1008), indicating the appropriateness of the synthetic conditions for the targeted samples preparation. It suggests that the introduction of a small number of Bi^3+^ and Eu^3+^ ions does not significantly alter the host structure. This observation may be attributed to the similarity in the ionic radii of Ca^2+^ ions (*r* = 1.12 Å, coordination number (CN) = 8) within the host and those of Bi^3+^ ions (*r* = 1.17 Å, CN = 8) and Eu^3+^ ions (*r* = 1.07 Å, CN = 8), where, the respective percentage differences are both 4.46% when these ions occupy the Ca^2+^ site. These weak impurity peaks at about 30.03°, 34.75°, 50.05°, and 59.42° for CTO:0.04Bi^3+^/0.20Eu^3+^ sample may be caused by the orthorhombic phase Ca_2_Ta_2_O_7_.

To better elucidate the effect of Bi^3+^ and Eu^3+^ doping ions on the microstructure of the host, the Rietveld refinement of CTO, CTO:0.04Bi^3+^, CTO:0.06Eu^3+^, CTO:0.04Bi^3+^/0.06Eu^3+^ and CTO:0.04Bi^3+^/0.16Eu^3+^ samples were performed on GSAS-II software by using Ca_1.953_Ta_0.89_Nd_0.07_Zr_0.08_O_7_ (ICSD# 97420) as the initial structure model, as displayed in Fig. 1b−f. The unit cell parameters and indicator parameters obtained by the refinement were listed in Table 1. The refined results converge to *R*_wp_ = 9.23%/10.00%/10.00%/8.78%/9.44%, *R*_p_ = 7.14%/7.43%/7.34%/6.54%/7.14% for the respective samples, confirming the satisfaction of all detected diffraction peaks with the reflection condition. All samples crystallize in the hexagonal system with the space group *P*3_1_21. For CTO host, the cell parameters are determined to be *a* = *b* = 7.359 Å, *c* = 18.118 Å, *V* = 849.660 Å^3^. Comparatively, the doped samples exhibit subtle variations in their cell parameters, primarily attributed to the slight differences in the radii between Bi^3+^/Eu^3+^ and Ca^2+^ ions. Upon the introduction of Bi^3+^ ions alone, the lattice parameter *a* and volume *V* of the sample show a slight increase, while the *c* value experiences a minor decrease. Similarly, the introduction of Eu^3+^ ions alone leads to a decrease in the lattice parameter *c* and *V* of the sample. These findings align with the variances in the radii of the doping ions, and the expansion and contraction of the unit cell volume further corroborate the successful incorporation of Bi^3+^/Eu^3+^ ions into the lattice sites of the host. Moreover, under the synergistic effect of Bi^3+^/Eu^3+^ co-doping, the lattice parameter *c* and *V* of these samples demonstrate a gradual decrease trend, indicating that the doping ions facilitates the contraction of the CTO unit cell along the *c*-axis.

**Table 1:** Refinement information of CTO, CTO:0.04Bi^3+^, CTO:0.06Eu^3+^, CTO:0.04Bi^3+^/0.06Eu^3+^, and CTO:0.04Bi^3+^/0.16Eu^3+^ samples

Formula	CTO	CTO:0.04Bi^3+^	CTO:0.06Eu^3+^	CTO:0.04Bi^3+^/0.06Eu^3+^	CTO:0.04Bi^3+^/0.16Eu^3+^
Crystal system	Hexagonal	Hexagonal	Hexagonal	Hexagonal	Hexagonal
Space group	*P*3_1_21	*P*3_1_21	*P*3_1_21	*P*3_1_21	*P*3_1_21
*Z*	6	6	6	6	6
*a* (Å)	7.359	7.362	7.359	7.362	7.362
*c* (Å)	18.118	18.112	18.105	18.077	18.055
*α* (° )	90.00	90.00	90.00	90.00	90.00
*γ* (° )	120.00	120.00	120.00	120.00	120.00
*V* (Å^3^)	849.660	850.052	849.072	848.377	847.447
*R*_wp_ (%)	9.23	10.0	10.0	8.78	9.44
*R*_exp_ (%)	4.53	4.59	4.41	4.29	4.75
*χ*^2^	4.16	4.80	5.18	4.18	3.95

*R*_p_ profile factor, *R*_wp_ weighted profile factor, *R*_exp_ expected weighted profile factor

**Fig. 2:**
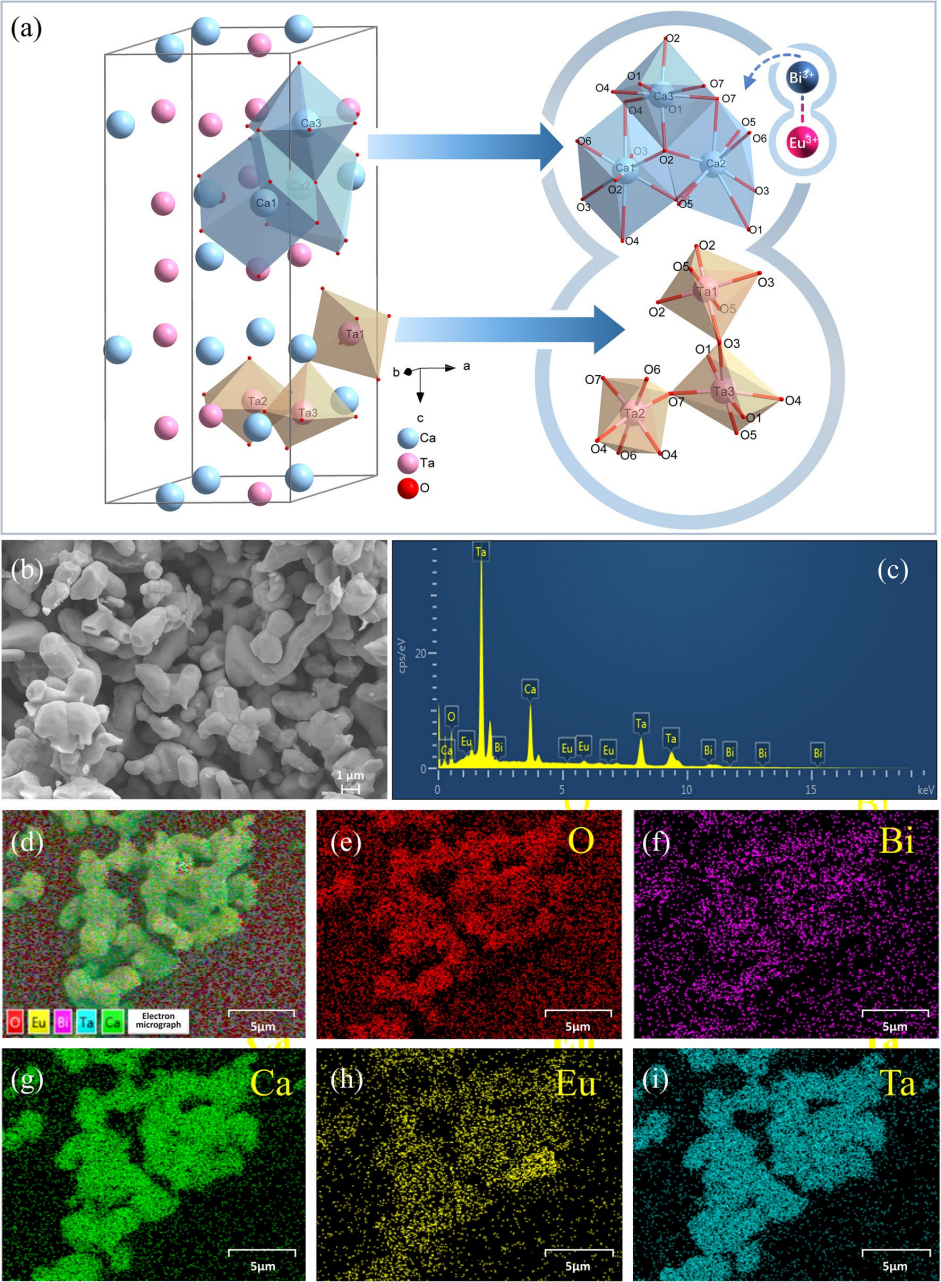
**a** Crystal structure of CTO. **b** SEM image and **c** EDS spectrum of CTO:0.04Bi^3+^/0.04Eu^3+^. **d** EDS layered image and elemental mapping images of **e** O, **f** Bi, **g** Ca, **h** Eu and **i** Ta

The schematic diagram of unit cell crystal structure for CTO host based on the refinement results was depicted in Fig. 2a. In this lattice, cubane [Ca1O_8_] and deformed dodecahedra [Ca2O_8_, Ca3O_8_] located at the positions of the 3a, 6c, and 3b Wyckoff lattices are connected by common edges. While the [Ta1O_6_, Ta2O_6_, Ta3O_6_] octahedrons are connected by a common vertex. In this context, the accommodation of Bi^3+^ and Eu^3+^ ions within the three Ca^2+^ cation sites is attributed to the marked disparity in ionic radii between Bi^3+^/Eu^3+^ and Ta^5+^ ions.

The morphology and size of phosphor take a crucial role in the performance of LED devices and temperature sensors. Typically, the particles with good crystallinity and regular morphology exhibit higher brightness and lower light attenuation when encapsulating LED devices [58]. Moreover, the use of smaller particle sizes can significantly augment thermal responsiveness, facilitating more precise temperature sensing. Thus, thorough investigation and fine-tuning of the characteristics of phosphors are essential to explore their potential applications in these domains. The SEM image of the CTO:0.04Bi^3+^/0.04Eu^3+^ sample prepared is depicted in Fig. 2b. The sample consists of smooth particles with sizes ranging from 0.8 to 3.5 μm. Furthermore, the elemental composition and homogeneity of the sample were confirmed through corresponding EDS and elemental mapping images, as depicted in Fig. 2c−i. The EDS spectrum unambiguously demonstrates the presence of exclusively Ca, Ta, Bi, Eu, and O elements in this sample, without any impurities detected. Additionally, the individual mapping images of the relevant elements reveal a uniform distribution throughout the sample's surface, indicating the successful synthesis of the target product.

**Fig. 3:**
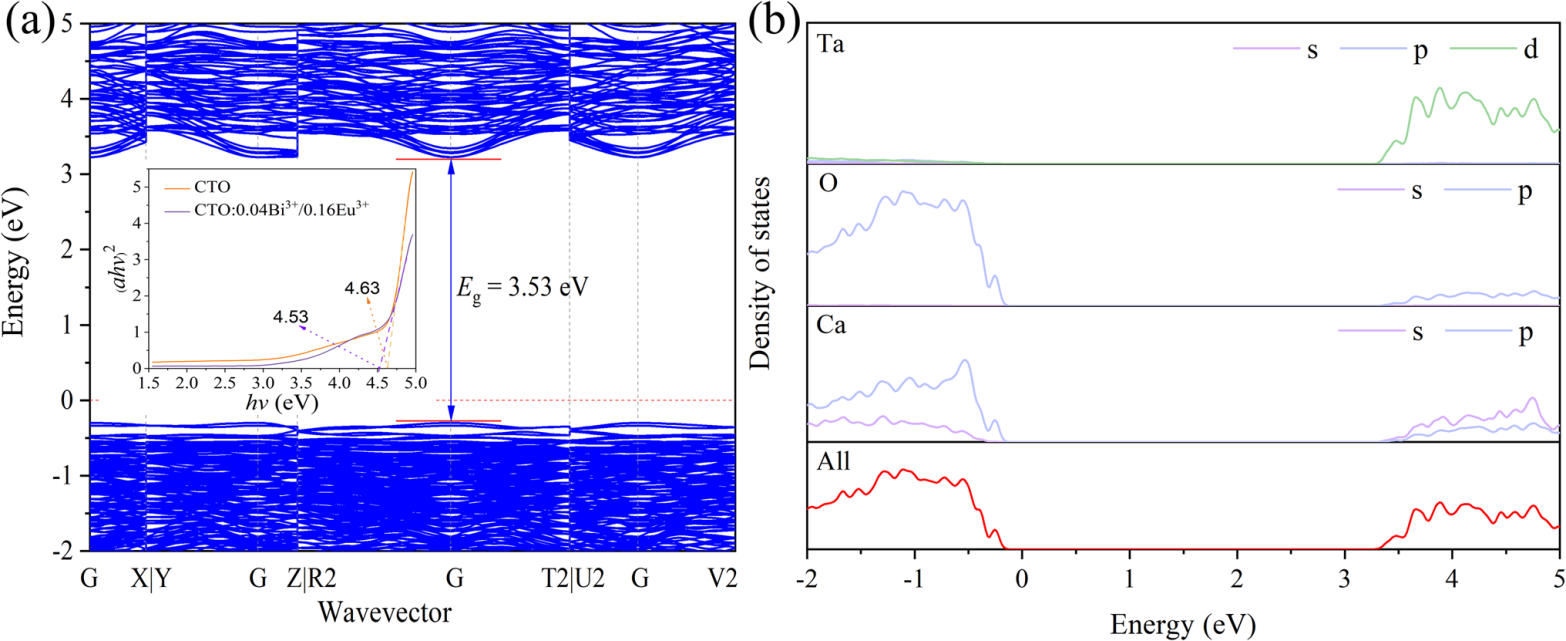
**a** Calculated band structure of CTO and **b** CTO density of states and partial density of states

Using the density functional theory (DFT) method, the electronic band structure of CTO was computed, and the findings were presented in Fig. 3a. The proximity of the valence band maximum (VBM) and conduction band minimum (CBM) at the same k-point confirms that CTO is a direct band gap material with a calculated bandgap value of 3.53 eV, making it well-suited as a luminescent host. Due to the local density approximation (LDA), the bandgap value calculated by the DFT method tends to be lower than the experimental value of 4.63 eV. Additionally, Fig. 3b illustrates the electronic density of states of CTO, revealing that the CBM is composed of O 2p orbitals and the VBM is composed of Ta 5d orbitals. Hence, the band gap in CTO primarily arises from the transition of electrons from the O 2p state to the Ta 5d state. Overall, these computational results offer significant insights into the electronic properties of CTO, which are crucial for comprehending its electronic behavior and for the design and optimization of related devices.

### Luminescent properties

**Fig. 4:**
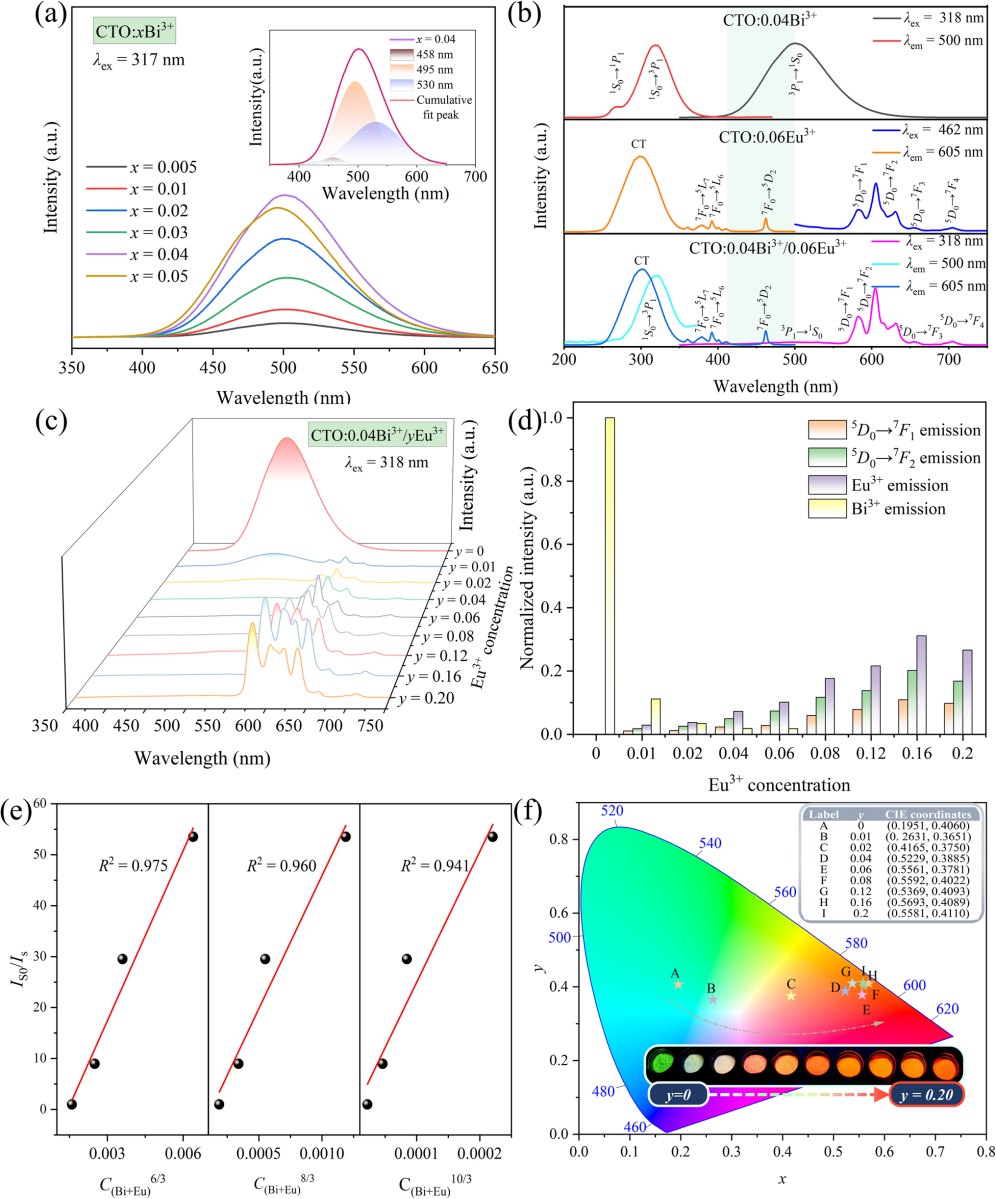
**a** PL spectra of CTO:*x*Bi^3+^. **b** PLE and PL spectra of CTO:0.04Bi^3+^, CTO:0.06Eu^3+^, and CTO:0.04Bi^3+^/0.06Eu^3+^ phosphors. **c** PL spectra of CTO:0.04Bi^3+^/*y*Eu^3+^ samples. **d** Histogram of the emission intensity of Bi^3+^ and Eu^3+^. **e** Relationship between *I*_S0_/*I*_S_ of Bi^3+^ and *C*_(Bi + Eu)_^*n*/3^ (*n* = 6, 8, 10). **f** CIE coordinates diagram and luminescent images of CTO:0.04Bi^3+^/*y*Eu^3+^ samples

Figure 4a describes the PL spectra of the CTO:*x*Bi^3+^ phosphors excited at 317 nm, and all these samples exhibit broad emission peaks in the 400−600 nm range with the maximum intensity observed at 500 nm, corresponding to the ^3^*P*_1_→^1^*S*_0_ transition of Bi^3+^ ion [59]. With the increase of Bi^3+^ doping concentration, the emission intensity of CTO:*x*Bi^3+^ samples increases gradually, reaching a peak of intensity at *x* = 0.04, follows by a decline attributed to non-radiative transitions resulting from the decreased distance between Bi^3+^ ions. Notably, the broadband peak of the optimal sample, CTO:0.04Bi^3+^, could be deconvolved into three distinct peaks located at 458, 495, and 530 nm, respectively. This observation suggests that Bi^3+^ ions occupy three distinct Ca sites (Ca1, Ca2 and Ca3) within the CTO host, a finding consistent with the crystal structure analysis presented in Fig. 2. Additionally, the lifetime fitting at different monitoring wavelengths in Fig. S2 also validates this conclusion.

To preliminarily assess the presence of energy transfer (ET) in the CTO:Bi^3+^/Eu^3+^ system, the PLE and PL spectra of CTO:0.04Bi^3+^, CTO:0.06Eu^3+^, and CTO:0.04Bi^3+^/0.06Eu^3+^ phosphors were presented in Fig. 4b for comparison. Within the range of 250−380 nm, the PLE spectrum of CTO:0.04Bi^3+^ reveals a broad excitation band alongside a faint band, corresponding to the ^1^*S*_0_ → ^3^*P*_1_ and ^1^*S*_0_ → ^1^*P*_1_ transitions of Bi^3+^ ions, respectively. Specifically, when excited at the maximum peak of 318 nm, a conspicuous bright green broadband emission at 500 nm can be observed, attributed to the ^3^*P*_1_→^1^*S*_0_ transition of Bi^3+^ ions. By analogy, the PLE spectrum of CTO:0.06Eu^3+^ sample showcases a wide Eu^3+^-O^2−^ charge transfer (CT) band spanning from 240 to 350 nm, accompanied by several sharp peaks ranging from 353 to 473 nm, emanating from the characteristic transitions of Eu^3+^ ions. Upon excitation at 462 nm, the PL spectrum exhibits multiple emission lines between 550 and 750 nm, corresponding to Eu^3+^: ^5^*D*_0_→^7^*F*_1_/^7^*F*_2_/^7^*F*_3_/^7^*F*_4_ transitions, respectively [60]. Additionally, a partial overlap between the PL spectrum of CTO:0.04Bi^3+^ and the PLE spectrum of CTO:0.06Eu^3+^ has been observed (indicated by the light blue background in Fig. 4b), hinting at the potential occurrence of an ET process between Bi^3+^ and Eu^3+^ ions. At the monitored wavelengths of 500 and 605 nm, respectively, the PLE spectra of the CTO:0.04Bi^3+^/0.06Eu^3+^ sample exhibit similar shapes to those of CTO:0.04Bi^3+^ and CTO:0.06Eu^3+^, respectively, resembling the behavior observed in Bi^3+^ and Eu^3+^ doped alone. Furthermore, under the excitation at 318 nm, characteristic transition peaks of Bi^3+^ and Eu^3+^ ions in the PL spectra of CTO:0.04Bi^3+^/0.06Eu^3+^ emerge concurrently. Notably, the intensity of the ^3^*P*_1_→^1^*S*_0_ transition of Bi^3+^ ions show a significant reduction in comparison to the one of CTO:0.04Bi^3+^, indicating a possible occurrence of ET between Bi^3+^ and Eu^3+^ ions.

To further substantiate the ET process occurring between Bi^3+^ and Eu^3+^ ions, a series of CTO:0.04Bi^3+^/*y*Eu^3+^ PL spectra were conducted and depicted in Fig. 4c. Additionally, the variation in luminescence intensities of Bi^3+^ and Eu^3+^ with increasing Eu^3+^ doping concentration is succinctly illustrated in Fig. 4d. Notably, a discernible contrast in intensities is observed, the Bi^3+^ intensity undergoes a sharp decline while the Eu^3+^ intensity gradually increases, reaching its peak at an Eu^3+^ concentration of 0.16. This pivotal observation serves as preliminary evidence for the Bi^3+^→Eu^3+^ ET process. Typically, non-radiative ET process entails electrical multipolar interaction or exchange interaction between sensitizer and activator ions, with exchange interaction occurring when the critical distance (*R*_c_) between the doped ions is less than 5 Å [61]. As for the CTO:0.04Bi^3+^/0.16Eu^3+^ phosphor, its specific parameters including *V*, *X*_c_, and *N* boast values of 847.447 Å^3^, 0.16, and 6, respectively. According to Blasse’s theory [62], the determined *R*_c_ is 11.05 Å, significantly surpassing the 5 Å threshold, indicating that electrical multipolar interaction predominantly contributes to the Bi^3+^→Eu^3+^ ET process.

On the basis of Dexter’s theory [63], the nature of the multipolar interaction between Bi^3+^ and Eu^3+^ ions can be distinguished using the following equation,

1 $$\frac{{I}_{S0}}{{I}_{S}}{\propto C}^{n/3},$$

where *C* represents the total concentration of Bi^3+^ and Eu^3+^. In this context, *n* = 6, 8, and 10 correspond to dipole-dipole, dipole-quadrupole, and quadrupole-quadrupole interactions, respectively. Figure 4e illustrates the results of linear regression analysis of the fitting points between *I*_*S*0_/*I*_*S*_ and *C*^*n*/3^. Notably, the best linear fit occurs when *n* = 6, yielding an *R*^2^ value of 0.975, confirming that the ET mechanism in the CTO:0.04Bi^3+^/*y*Eu^3+^ phosphors is primarily governed by a dipole-dipole interaction. As illustrated in Fig. 4f, the CIE chromaticity coordinates of the CTO:0.04Bi^3+^/*y*Eu^3+^ phosphors exhibit a pronounced shift from (0.195, 0.406) to (0.558, 0.411) as the concentration of Eu^3+^ increased from *y* = 0 to *y* = 0.20, which results in a notable change in the luminescence color of these phosphors, gradually shifting from green region toward reddish-orange region. Remarkably, this color evolution aligns perfectly with the observed luminescent images. It is worth noting that this change in emission color can be purposefully manipulated by precisely controlling the quantity of Eu^3+^ in the sample. This controllability holds immense potential for various applications, particularly in multicolor devices where the emission color needs to be tailored to specific requirements.

### Energy transfer analysis

**Fig. 5:**
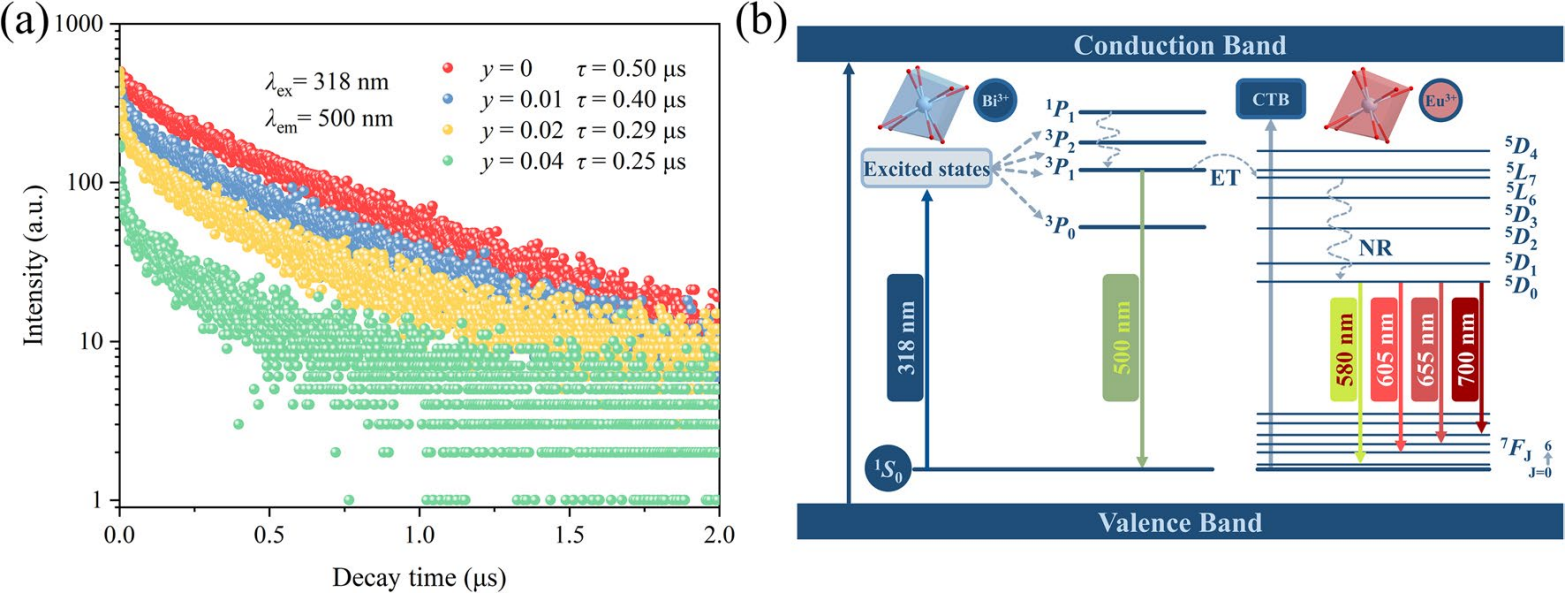
**a** Decay curves of CTO:0.04Bi^3+^/*y*Eu^3+^ (*λ*_ex_ = 318 nm, *λ*_em_ = 500 nm). **b** Energy transfer model of CTO:0.04Bi^3+^/Eu^3+^

When the value of *y* exceeds 0.06 in CTO:0.04Bi^3+^/*y*Eu^3+^ samples, the characteristic emissions of Bi^3+^ are practically negligible. Therefore, the decay curves of CTO:0.04Bi^3+^/*y*Eu^3+^ (*y* = 0, 0.01, 0.02, and 0.04) samples under 318 nm excitation were tested to validate the ET process in the system, as depicted in Fig. 5a. The decay curves of Bi^3+^ ions, monitored at 500 nm, were suitably modeled using a bi-exponential function [64]:

2 $$I={{I}_{0}\text{+}{A}}_{1}\text{exp}\left(-\frac{t}{{\tau }_{1}}\right)+{A}_{2}{\text{exp}}\left(-\frac{t}{{\tau }_{2}}\right).$$

Here, *I*_0_ means the initial intensity, *A*_1_ and *A*_2_ are constants, and *τ*_1_ and *τ*_2_ present the decay times. The average lifetime (*τ*) could be estimated by the following approach [65]:

3 $$\tau \text{=}{\text{(}{A}}_{1}{{\tau }_{1}}^{2}\text{+}{A}_{2}{{\tau }_{2}}^{2}\text{)/(}{A}_{1}{\tau }_{1}\text{+}{A}_{2}{\tau }_{2}\text{).}$$

Upon increasing the concentration of Eu^3+^ ions from 0 to 0.04, the average lifetime of Bi^3+^ decreases from 0.50 to 0.25 μs gradually, which serves as a further confirmation of the ET occurring between Bi^3+^ and Eu^3+^.

To visually illustrate the ET route between Bi^3+^ and Eu^3+^, a simplified ET model is employed, as depicted in Fig. 5b. Initially, under 318 nm excitation, electrons in the ground state ^1^*S*_0_ of Bi^3+^ absorb energy and become excited, transitioning to the ^3^*P*_1_ excited state. Subsequently, most of these excited electrons undergo radiative transitions, and then come back to the ground state ^1^*S*_0_, thereby generating a wide green emission band centered at 500 nm. However, the others could migrate to the ^5^*L*_6_ energy level of Eu^3+^ through the ET process because of the closely matched energy levels. Then, these electrons relax to the ^5^*D*_0_ state by non-radiative relaxation. Eventually, the electrons fall into various ^7^*F*_*J*_ (*J* = 1, 2, 3, and 4) energy levels via radiative transitions accompanied by the characteristic emission of Eu^3+^ ions.

### Luminescence thermometry based on FIR technology

**Fig. 6:**
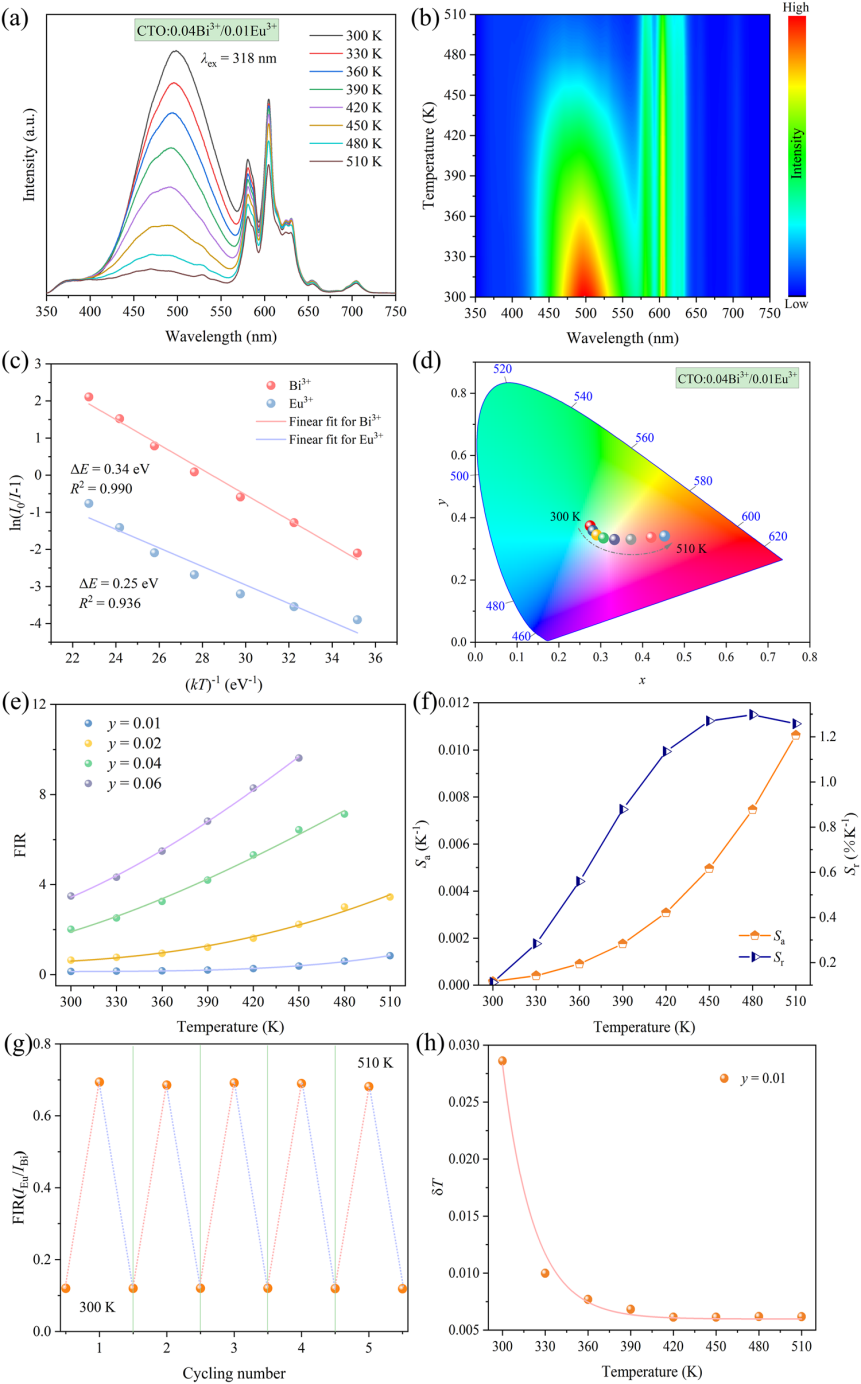
**a** Temperature-dependent PL spectrum, **b** contour map, **c** linear fitting of ln(*I*_0_/*I−*1) versus (*kT*)^−1^, **d** CIE chromaticity diagram at different temperatures of CTO:0.04Bi^3+^/0.01Eu^3+^. **e** Evolution of FIR with temperature for CTO:0.04Bi^3+^/*y*Eu^3+^. **f** *S*_a_ and *S*_r_ versus absolute temperature, **g** cycling measurement, and **h** temperature resolution of CTO:0.04Bi^3+^/0.01Eu^3+^

The potential application of FIR-based technology in temperature sensing and optical thermometry of the prepared samples was demonstrated through the thermal evolution PL spectra and contour maps of CTO:0.04Bi^3+^/*y*Eu^3+^ (*y* = 0.01, 0.02, 0.04, and 0.06) featured in Fig. 6a, b and Fig. S3a−f. As the temperature increases, distinctly different TQ behaviors are observed for Bi^3+^ and Eu^3+^. Taking CTO:0.04Bi^3+^/0.01Eu^3+^ samples as an example, within the range of 300−510 K, Bi^3+^ ions exhibit a pronounced temperature response in CTO materials, resulting in a substantial decrease in luminous intensity. In contrast, Eu^3+^ ions present better thermal stability, with both ions maintaining relatively unchanged emission peak positions. It is well known that the greater the disparity in the TQ behavior between Bi^3+^ and Eu^3+^, the higher the FIR value as a signal reflecting the optical temperature measurement characteristics. The remarkable variation can be attributed to the different quenching mechanisms of Bi^3+^ and Eu^3+^ ions. Specifically, the TQ of Bi^3+^ ions primarily arises from the cross-relaxation between the energy levels of ^1^*S*_0_ and ^3^*P*_1_, whereas the one of Eu^3+^ ions is mainly due to the substantial energy difference between ^7^*F*_6_ and ^5^*D*_0_ levels, involving multi-phonon deexcitation as the primary mechanism [66].

Furthermore, the activation energy (Δ*E*) of CTO:0.04Bi^3+^/*y*Eu^3+^ (*y* = 0.01, 0.02, 0.04, and 0.06) samples were estimated by using the Arrhenius-type activation model to verify the TQ mechanism [67]:

4 $$\text{ln}\left(\frac{{I}_{0}}{I}-1\right)=\text{ln}A-\frac{\Delta E}{{k}_{\text{B}}T},$$

where *I*_0_ and *I* are the PL intensities of CTO:0.04Bi^3+^/*y*Eu^3+^ at 300 K and different operating temperatures *T*, respectively. *A* is a constant, *k*_B_ means Boltzmann’s constant. According to the above formula, the Δ*E* values of the CTO:0.04Bi^3+^/0.01Eu^3+^ samples are 0.34 and 0.25 eV, respectively. The corresponding *E*_a_ values for other samples can be found in Fig. S4a−c. These distinct Δ*E* values further confirm the differential TQ mechanisms of Bi^3+^ and Eu^3+^ ions. Moreover, when the temperature ranges from 300 to 510 K, the CTO:0.04Bi^3+^/0.01Eu^3+^ sample exhibits a color transformation from blue-green to light yellow, ultimately transitioning to purplish red, as demonstrated in Fig. 6d. Further variations in the emitting color of CTO:0.04Bi^3+^/*y*Eu^3+^ (*y* = 0.02, 0.04, and 0.06) samples at different temperatures were depicted in Fig. S3g−i. This fascinating behavior indicates the potential use of the prepared samples for visualized thermometer or serving as high-temperature safety markings.

The function relations of FIR and temperature could be described by the Strike and Fonger theories [68]:

5 $$\text{FIR}=\frac{{I}_{\text{Eu}}}{{I}_{\text{Bi}}}=\frac{\int {I}_{\text{Eu}}(\lambda ,T)\text{d}\lambda }{\int {I}_{\text{Bi}}(\lambda ,T)\text{d}\lambda }\approx A+B\text{exp}\left(-\frac{\Delta E}{kT}\right),$$

where *A* and *B* present the corresponding parameters. Figure 6e exhibits the variation in FIR value of CTO:0.04Bi^3+^/*y*Eu^3+^ (*y* = 0.01, 0.02, 0.04, and 0.06) phosphors as a function of inverse absolute temperature. The emission peaks of Bi^3+^ and Eu^3+^ ions, ranging in 453−512 and 600−610 nm, respectively, were utilized for calculation. It is evident that the FIR values of the CTO:0.04Bi^3+^/0.06Eu^3+^ sample exhibit the most rapid change as the temperature increases, reaching a maximum of 9.63 at 450 K.

In practical applications, the evaluation of optical thermometry relies on two important parameters, absolute sensitivity (*S*_a_) and relative sensitivity (*S*_r_). These parameters can be assessed using the following formulas:

6 $${S}_{\text{a}}=\left|\frac{\partial \text{FIR}}{\partial T}\right|=B\text{exp}\left( -\frac{\Delta E}{kT}\right)\times \frac{\Delta E}{{kT}^{2}},$$

7 $${S}_{\text{r}}=100\text{\%}\times \left|\frac{1}{\text{FIR}}.\frac{\partial \text{FIR}}{\partial T}\right|=100\text{\%}\times \frac{B\text{exp}(-\frac{\Delta E}{kT})}{B\text{exp}(-\frac{\Delta E}{kT})+A}\times \frac{\Delta E}{k{T}^{2}}.$$

For the series samples of CTO:0.04Bi^3+^/*y*Eu^3+^ (*y* = 0.01, 0.02, 0.04, and 0.06), *S*_a_ consistently demonstrates an ascending trend, whereas the behavior of *S*_r_ varies across these samples. Specifically, for *y* = 0.01 and 0.02, *S*_r_ undergoes an initial increase, reaches a peak, and subsequently experiences a gradual decline. Conversely, the *S*_r_ of these samples with *y* values of 0.04 and 0.06 demonstrates a decrease as the temperature rises, as depicted in Fig. 6f and Fig. S4d−f. The maximum *S*_a_/*S*_r_ values for CTO:0.04Bi^3+^/*y*Eu^3+^ (*y* = 0.01, 0.02, 0.04, and 0.06) were found to be 0.011 (at 510 K)/1.298% K^−1^ (at 480 K), 0.026 (at 510 K)/0.937% K^−1^ (at 390 K), 0.036 (at 480 K)/1.084 K^−1^ (at 300 K), and 0.055 (at 510 K)/0.846%^−1^ (at 300 K), respectively. These outcomes unequivocally demonstrate the substantial influence of doping concentrations on the sensitivity of temperature sensing. It is noteworthy that the maximum *S*_a_ and *S*_r_ values of CTO:0.04Bi^3+^/0.04Eu^3+^ phosphor are comparable to most of those phosphors co-doped with Bi^3+^ and Eu^3+^ ions reported previously based on FIR technology, as listed in Table 2. Furthermore, the temperature cycling performance of the FIR (*I*_Eu_/*I*_Bi_) for CTO:0.04Bi^3+^/0.01Eu^3+^ within the range of 300−510 K is depicted in Fig. 6g. Following five cycles of heating and cooling, the FIR exhibits minimal changes, affirming the excellent repeatability of the prepared phosphors. Paired with a comparative analysis of the XRD patterns for the CTO:0.04Bi^3+^/0.01Eu^3+^ phosphor before and after five cycles, as presented in Fig. S5. The largely unaltered structure serves as additional evidence of the remarkable thermal stability exhibited by the sample.

**Table 2:** Maximum *S*_a_ and *S*_r_ values, excitation wavelength, temperature sensing ranges of optical thermometers

Compounds	*λ*_ex_ (nm)	Max. *S*_a_ (K^−1^)	Max. *S*_r_ (% K^−1^)	Temperature range (K)	Refs.
Sr_3−*x*_Gd_*x*_GaO_4 +__*x*_F_1−*x*_:Bi^3+^, Eu^3+^	235	0.067	1.27	298−523	[25]
SrY_2_O_4_:Bi^3+^,Eu^3+^	330	0.0433	0.86	313−563	[71]
Sr_2.585_Gd_0.4_AlO_4.4_F_0.6_:Bi^3+^, Eu^3+^	315	0.0064	1.17	303−523	[66]
La_2_MgTiO_6_:Dy^3+^, Mn^4+^	350	0.022	2.622	303−563	[72]
LaScO_3_:Bi^3+^, Eu^3+^	308	0.118	0.795	280−480	[73]
La_2_MgTiO_6_:Mn^4+^, Eu^3+^	328	0.058	2.09	300−500	[74]
GdNbO_4_:Bi^3+^, Eu^3+^	308	0.0367	3.81	303−523	[46]
Ca_2_LaTaO_6_:Bi^3+^, Eu^3+^	266	0.041	0.76	293−510	[47]
CTO:0.04Bi^3+^/0.01Eu^3+^	318	0.011	1.298	300−510	This work
CTO:0.04Bi^3+^/0.06Eu^3+^	318	0.055	0.846	300−510	This work

Moreover, the temperature resolution (*δ*_T_), as an important parameter used in optical thermometers to distinguish small temperature variations, can be derived from the following equation [69, 70]:

8 $${\delta }_{\text{T}}=\frac{1}{{S}_{\text{r}}}\frac{{\delta }_{\text{FIR}}}{\text{FIR}},$$

where *δ*_FIR_ represents the standard deviation of FIR. In the case of CTO:0.04Bi^3+^/0.01Eu^3+^ phosphor, the optimal value of *δ*_T_ is remarkably low, measuring only 0.0286 K at 298 K. This value surpasses the practical application requirements, as illustrated in Fig. 6h.

### Application for WLED

**Fig. 7:**
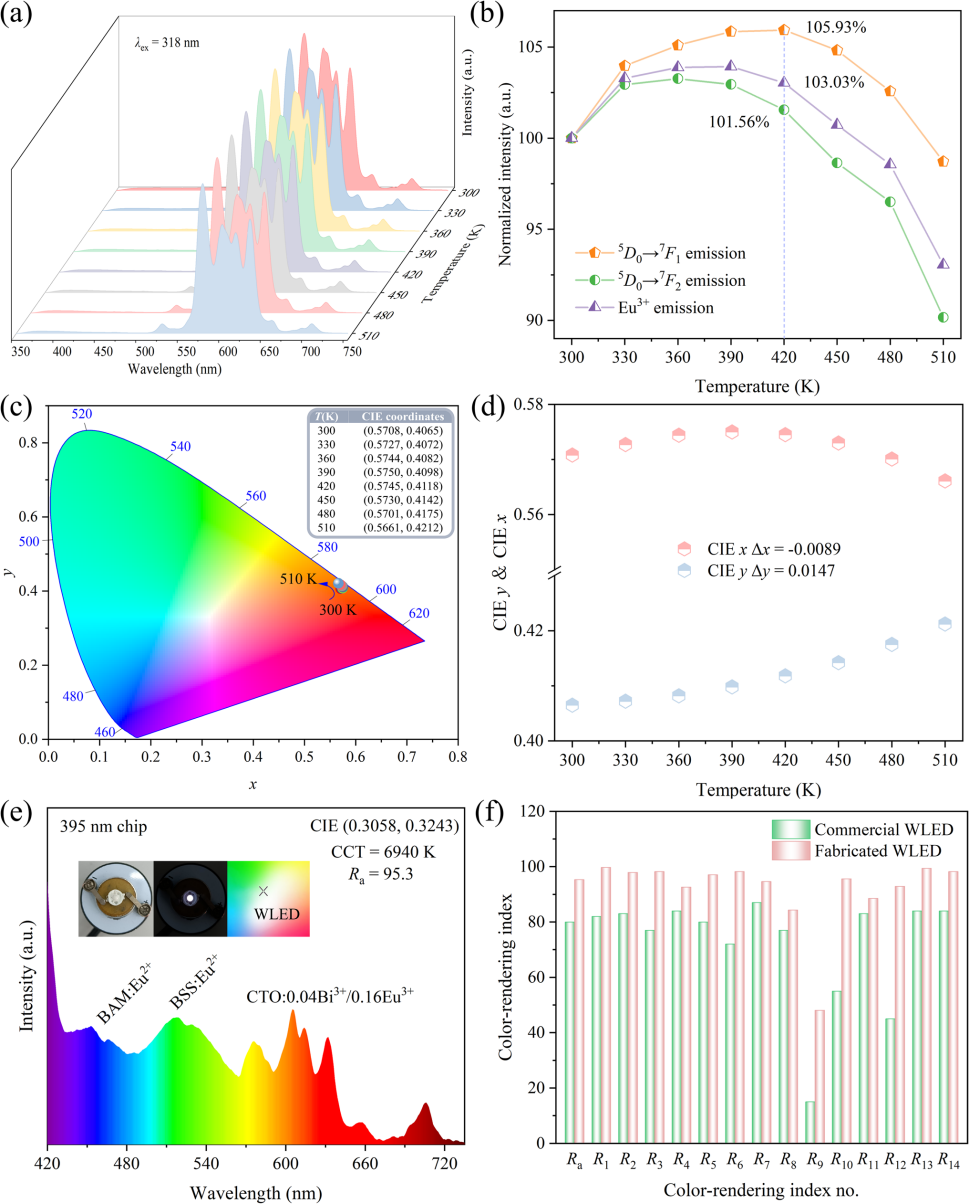
**a** Temperature-dependent PL spectra, **b** normalized PL intensity based on various temperatures, **c** CIE chromaticity diagram at different temperatures, **d** relationship between CIE *x*, CIE *y*, and temperature of CTO:0.04Bi^3+^/0.16Eu^3+^. **e** EL spectrum of the constructed WLED and **f** histogram of the comparison of the CRI index of constructed WLED and commercial one

Aside from the exceptional multi-color tunable luminescence and precise optical temperature measurement capabilities discussed previously, the remarkable luminescent characteristics of CTO:0.04Bi^3+^/0.16Eu^3+^ phosphor indicate its potential application in WLED devices. Commonly, LEDs can generate elevated temperatures of approximately 420 K during operation. To comprehensively assess the luminescent properties and color stability of this phosphor under such high temperatures, temperature-dependent PL spectra of CTO:0.04Bi^3+^/0.16Eu^3+^ sample were scrutinized meticulously, as depicted in Fig. 7a. Evidently, no significant alterations are observed in the shape or position of the characteristic emission peaks of Eu^3+^ ions across varying temperatures.

Furthermore, both primary emission peaks (^5^*D*_0_→^7^*F*_1_ and ^5^*D*_0_→^7^*F*_2_) of Eu^3+^ ions at each temperature, alongside the cumulative emission intensities within the 500−750 nm range, have been integrated and normalized meticulously, as illustrated in Fig. 7b. Surprisingly, the CTO:0.04Bi^3+^/0.16Eu^3+^ sample exhibits a remarkable zero-thermal-quenching behavior upon reaching 420 K, with the integrated intensities of the respective emissions maintaining at 105.93%, 101.56%, and 103.03% of their initial levels, respectively, follows by a gradual decrease as temperature rises. Notably, the quicker escalation in the integrated intensity of ^5^*D*_0_→^7^*F*_1_ compared to that of ^5^*D*_0_→^7^*F*_2_ indicates a reduction in the asymmetry ratio (*R*) of *I*_ED_/*I*_MD_ with increasing temperature, signifying diminishing distortion around the Eu^3+^ ions and connoting an ascent in sample stability with rising temperature. Therefore, the CTO:0.04Bi^3+^/0.16Eu^3+^ phosphor, boasting excellent thermal stability, holds promise for application in high-power LED devices. Furthermore, phosphors exhibiting high IQE hold substantial practical significance across a myriad of applications. Notably, the IQE of CTO:0.04Bi^3+^/0.16Eu^3+^ at 318 nm excitation is calculated to be as remarkable as 58.4% (as visually depicted in Fig. S6).

The zero-thermal-quenching phenomenon has also been observed in CTO:0.04Bi^3+^/0.12Eu^3+^ and CTO:0.04Bi^3+^/0.20Eu^3+^ samples, with a more pronounced effect as the Eu^3+^ doping concentration increases. For instance, at 420 K, the cumulative emission intensities of Eu^3+^ in these two samples still remain at 101.96% and 105.15% of their respective initial levels respectively, as demonstrated in Fig. S7. This result highlights the enhanced stability and resistance to TQ exhibited by the phosphors with higher Eu^3+^ doping concentrations. The zero-thermal-quenching behavior observed in the CTO:0.04Bi^3+^/*y*Eu^3+^ (*y* = 0.12, 0.16, and 0.20) samples may be related to the temperature-induced redshift of CTB resulting in an abnormal thermal quenching at the edge, which called edge abnormal thermal quenching (EATQ), and has also been observed in Gd_3_TaO_7_:Eu^3+^ [75], CaMoO_4_:Er^3+^/Eu^3+^ and LuVO_4_:Eu^3+^ [76]. The temperature-dependent PLE spectra for CTO:0.16Eu^3+^, CTO:0.04Bi^3+^/0.16Eu^3+^, and CTO:0.04Bi^3+^ have been illustrated in Fig. S8. With the increase of temperature, the positions of ^1^*S*_0_→^3^*P*_1_ and ^1^*S*_0_→^1^*P*_1_ transitions of Bi^3+^ in CTO:0.04Bi^3+^ do not shift significantly except for the obvious change in intensity. Whereas the CTB band of Eu-O in CTO:0.16Eu^3+^, CTO:0.04Bi^3+^/0.16Eu^3+^ showed a decreasing trend. The energy required for the transition of electrons from the ground state to CTB decreases, resulting in the redshift of CTB in the excitation spectrum, and the edge intensity of CTB at longer wavelengths gradually increases with the increase of temperature. In addition, the vibrations of multiple vibrational sublevels upon the ground state will intensify after temperature increases and thermal population for initial state will changes, resulting in zero thermal quenching of CTO: 0.04Bi^3+^/0.16Eu^3+^.

Coupled with the CIE coordinates (*x*, *y*) of CTO:0.04Bi^3+^/0.16Eu^3+^ obtained at varying temperatures, ranging from (0.5708, 0.4065) to (0.5661, 0.4212), the marginal disparity in CIE *x* and *y*, being only 0.0089 and 0.0147 respectively, attests to the excellent color stability of the material, as shown in Fig. 7c, d. The color coordinates (0.5708, 0.4065) at 300 K, derived from the variable temperature PL test, exhibit slight deviation from the values of (0.5693, 0.4089) for the CTO:0.04Bi^3+^/0.16Eu^3+^ sample presented in Fig. 4f. The tiny variance is likely attributable to the disparity in the molds employed at room temperature. Figure S9a, b displayed the XRD and PL plots of the CTO:0.04Bi^3+^/0.16Eu^3+^ sample before and after immersion in various pH (1.34 and 13.25) solutions for 24 h. Notably, the XRD diffraction peaks of the sample after immersion exhibit no significant changes compared to those prior to immersion, indicating the robust acid/alkali resistance of the prepared CTO:0.04Bi^3+^/0.16Eu^3+^. Surprisingly, the emission intensity of the sample after 24 h of immersion only shows a marginal decrease when contrasted with the unsoaked sample, underscoring its promising application potential in complex practical environments.

The EL spectrum of the fabricated WLED device, composed of BaMgAl_10_O_17_:Eu^2+^ (BAM:Eu^2+^, blue), (Ba,Sr)_2_SiO_4_:Eu^2+^ (BSS:Eu^2+^, green), and as-prepared CTO:0.04Bi^3+^/0.16Eu^3+^ phosphors with a 395 nm chip at 60 mA current and 3 V, was depicted in Fig. 7e. The resulting WLED emits a bright white light with CIE chromaticity coordinates (0.3058, 0.3243), positioned within the white light region. The color rendering index (CRI) value serves as a crucial indictor for evaluating the quality of WLEDs. A comparison between the full set of the CRIs and *R*_a_ of the fabricated WLED and commercial one (YAG:Ce^3+^ + blue chip) was presented in Fig. 7f. Clearly, all these parameters of the prepared WLED device, particularly the *R*_a_ (95.3), significantly surpass those of commercial counterpart (*R*_a_ = 80), highlighting its potential application in WLED technology [77]. Although the correlated color temperature (CCT) of the prepared WLED (6940 K) is relatively high, it still represents a noticeable improvement compared to the commercial one (7746 K).

## Conclusions

A series of multicolor tunable Ca_2_Ta_2_O_7_:Bi^3+^/Eu^3+^ (CTO:Bi^3+^/Eu^3+^) dual-emitting center optical thermometers have been designed and prepared through the high-temperature solid-state reaction method. Upon excitation at 318 nm, the obtained phosphors exhibit wide green emission bands of Bi^3+^ (^3^*P*_1_→^1^*S*_0_) and characteristic luminescence of Eu^3+^ (^5^*D*_0_→^7^*F*_*J*_, *J* = 0, 1, 2, 3, 4). By modulating the ratio of Bi^3+^/Eu^3+^ and utilizing the energy transfer from Bi^3+^ to Eu^3+^ ions, the multicolor tunable emission from green to reddish-orange was realized. The optical temperature measurement performance of CTO:0.04Bi^3+^/Eu^3+^ phosphors from 300 to 510 K were assessed based on the distinctly different TQ behaviors between Bi^3+^ and Eu^3+^. The maximum values of *S*_a_ and *S*_r_ reached 0.055 K^−1^ (at 510 K) and 1.298% K^−1^ (480 K), respectively. Moreover, the thermochromic behavior of the luminescence color with respect to temperature could be clearly observed, and the temperature cycling test shows the superior stability and repeatability, indicating that the prepared sample has a potential application prospect in visualized thermometer or high-temperature safety marking. Furthermore, due to the excellent zero thermal quenching performance, acid/alkali resistance and color stability of CTO:0.04Bi^3+^/0.16Eu^3+^ phosphor, a WLED device with *R*_a_ of 95.3 was constructed by combining the near-ultraviolet LED chip, CTO:0.04Bi^3+^/0.16Eu^3+^, commercially available blue and green phosphors, showcasing the potential application of CTO:0.04Bi^3+^/0.16Eu^3+^ in near-UV pumped WLED devices.

## Supplementary Information

Below is the link to the electronic supplementary material.

Supplementary file1 (PDF 1305 KB)

## Data Availability

The data that support the findings of this study are available from the corresponding author, upon reasonable request.
