# Supplementary material for: Dual-functional application of Ca2Ta2O7:Bi3+/Eu3+ phosphors in multicolor tunable optical thermometry and WLED
Source: Front Optoelectron. 2024 Sep 4;17(1):31. doi: 10.1007/s12200-024-00134-2 (PMC11374947; doi:10.1007/s12200-024-00134-2)
Supplement: Supplementary file 1 — Supplementary file1 (PDF 1305 KB) [file 12200_2024_134_MOESM1_ESM.pdf]

Supporting Information

Dual-functional application of $Ca_2Ta_2O_7:Bi^{3+}/Eu^{3+}$ phosphors in multicolor tunable optical thermometry and WLED

Jingjing Ru[a*], Bing Zhao[b*], Fan Zeng[a, c], Feiyun Guo[d], Jinhua Liu[e], Jianzhong Chen[d]

a. College of New Energy and Materials, Fujian Province University Key Laboratory of Green Energy and Environment Catalysis, Ningde Normal University, Ningde, Fujian 352100, China;

b. College of Mechanical and Electrical Engineering, Ningde Normal University, Ningde, Fujian 352100, China;

c. School of Environment and Resources, School of Carbon Neutral and Modern Industry, Fujian Normal University, Fuzhou, Fujian 350007, China;

d. College of Chemistry, Fuzhou University, Fuzhou, Fujian 350108, China;

e. School of Pharmacy and Medical Technology, Key Laboratory of Pharmaceutical Analysis and Laboratory Medicine of Fujian Province, Putian University, Putian, Fujian 351100, China.

*Corresponding authors

E-mail: jing3032357@163.com;woshizhaobing@outlook.com

1 LED fabrication

A WLED lamp was gained by connecting the tricolor phosphors as $BaMgAl_{10}O_{17}:Eu^{2+}$ ($BAM:Eu^{2+}$, blue), (Ba, Sr)$_2SiO_4:Eu^{2+}$ ($BSS:Eu^{2+}$, green), and the prepared CTO:0.04Bi^{3+}/0.16Eu^{3+} (red) with a 395 nm chip. The blue, green and red phosphors were mixed with the silicone gel according to the mass ratio of 3:1:8. Then the mixture were dropped on the 395 nm chip and dried in an oven at 110 °C for 1 h. The parameters of EL spectra, CIE coordinates, CCT, and R_a of the WLED device has been measured under a voltage of 3 V and a current of 10 to 120 mA.

2 Characterization

Powder X-ray diffraction (XRD) analysis was conducted using a Bruker D8 Advance diffractometer, with a scanning range of 10 – 80°(2θ). The microscopic morphology, elemental composition and distribution of the resultant sample were performed on a Thermo Scientific Apreo 2S scanning electron microscope (SEM). Luminescence properties, including photoluminescence excitation (PLE), photoluminescence (PL), and temperature-dependent PL spectra (300 K to 510 K) were acquired using a Hitachi F4700 fluorescence spectrometer. Decay lifetimes and internal quantum efficiency (IQE) were investigated employing an Edinburgh FLS 980 spectrophotometer. Electroluminescence (EL) spectrum of the assembled WLED device was determined with an HP9000 fiber optic spectrometer.

3 Computational calculation

The calculation of $Ca_2Ta_2O_7$ electronic structure was completed within the framework of density functional theory (DFT). In DFT calculations, $Ca_{24}Ta_{24}O_{84}$ bulk structure was constructed by expanding $Ca_2Ta_2O_7$ unit cell into 2×1×1 supercell. Structural optimizations were performed by Vienna *Ab-initio* Simulation Package (VASP)[1] with the projector augmented wave (PAW) method.[2] The exchange-functional was treated using the Perdew-Burke-Ernzerhof (PBE)[3] functional, in combination with the DFT-D3 correction.[4] Cut-off energy of the plane-wave basis was set as 450 eV. For optimization of lattice size $Ca_{24}Ta_{24}O_{84}$ bulk structure, the Brillouin zone integration was performed with a Monkhorst-Pack[5] *k*-point sampling of 2×4×1. The self-consistent calculations applied a convergence energy threshold of 10^{-5} eV. The equilibrium geometries and lattice constants were optimized with maximum stress on each atom within 0.02 eV Å^{-1}. Band structure and density of state of $Ca_{24}Ta_{24}O_{84}$ were calculated by GGA-PBE functional and exported by vaspkit interface.[6]

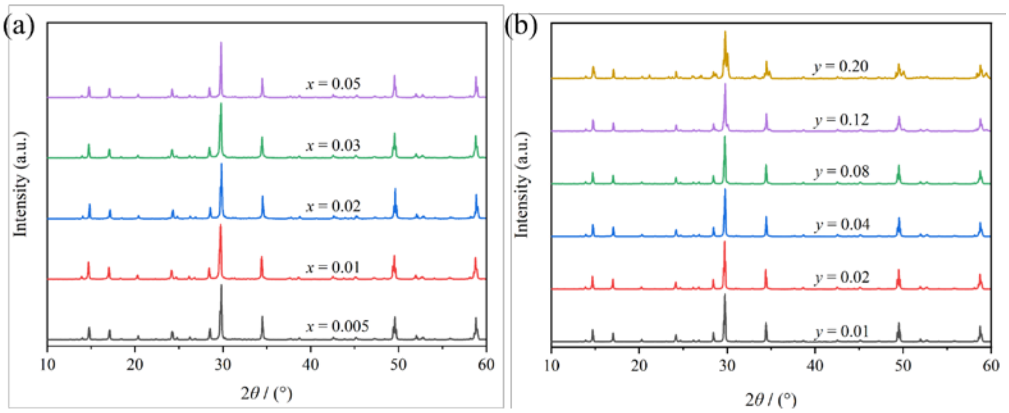

Fig. S1 The XRD patterns of CTO:xBi^{3+} (a) and CTO:0.04Bi^{3+}/yEu^{3+} (b).

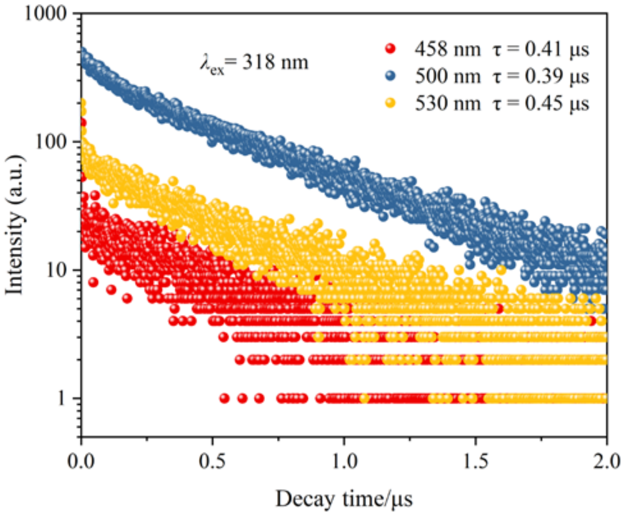

Fig. S2 The decay curves of CTO:0.04Bi^{3+} at 458, 500 and 530 nm respectively.

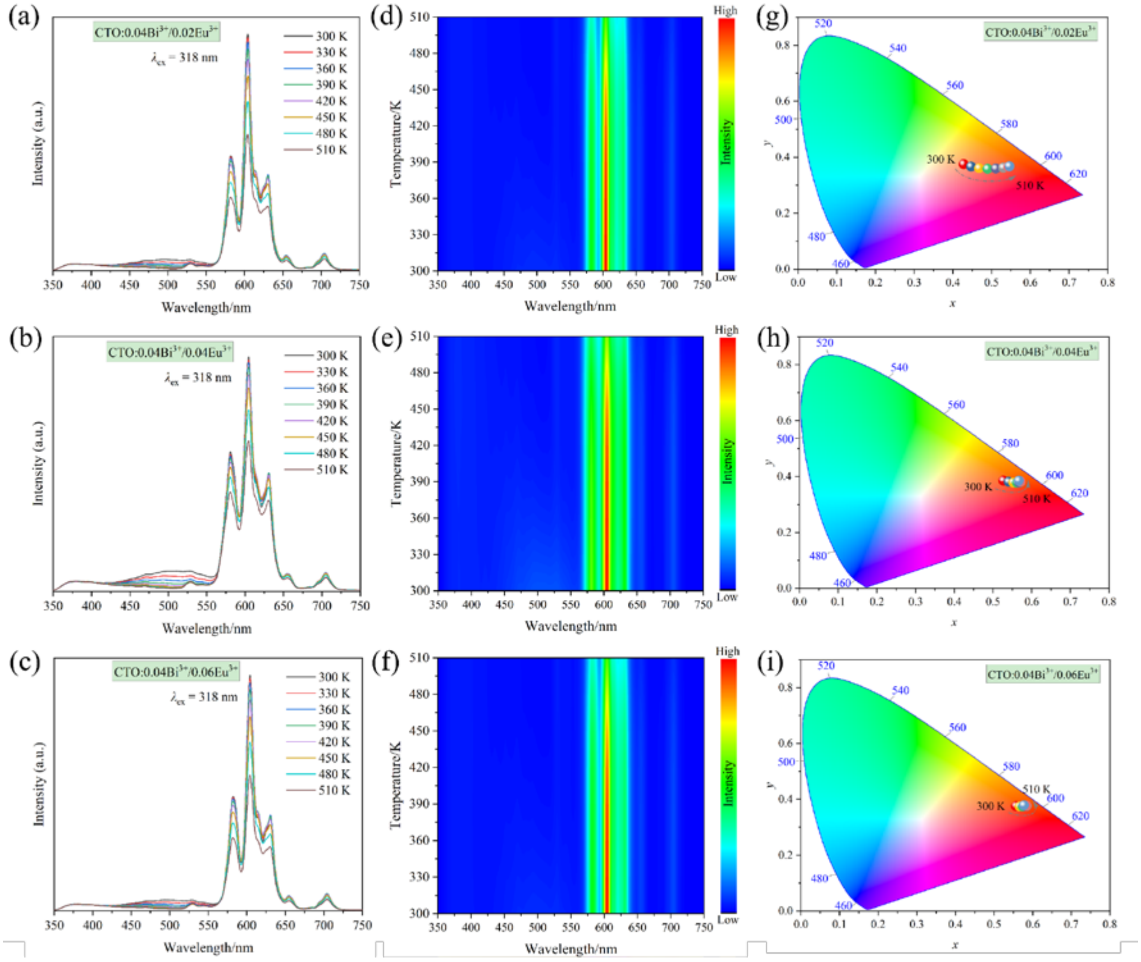

Fig. S3 Temperature-dependent PL spectra (a-c), contour maps (d-f), and CIE chromaticity diagram at different temperatures (g-i) of CTO:0.04Bi^{3+}/$y$$Eu^{3+}$ (y = 0.02, 0.04, 0.06).

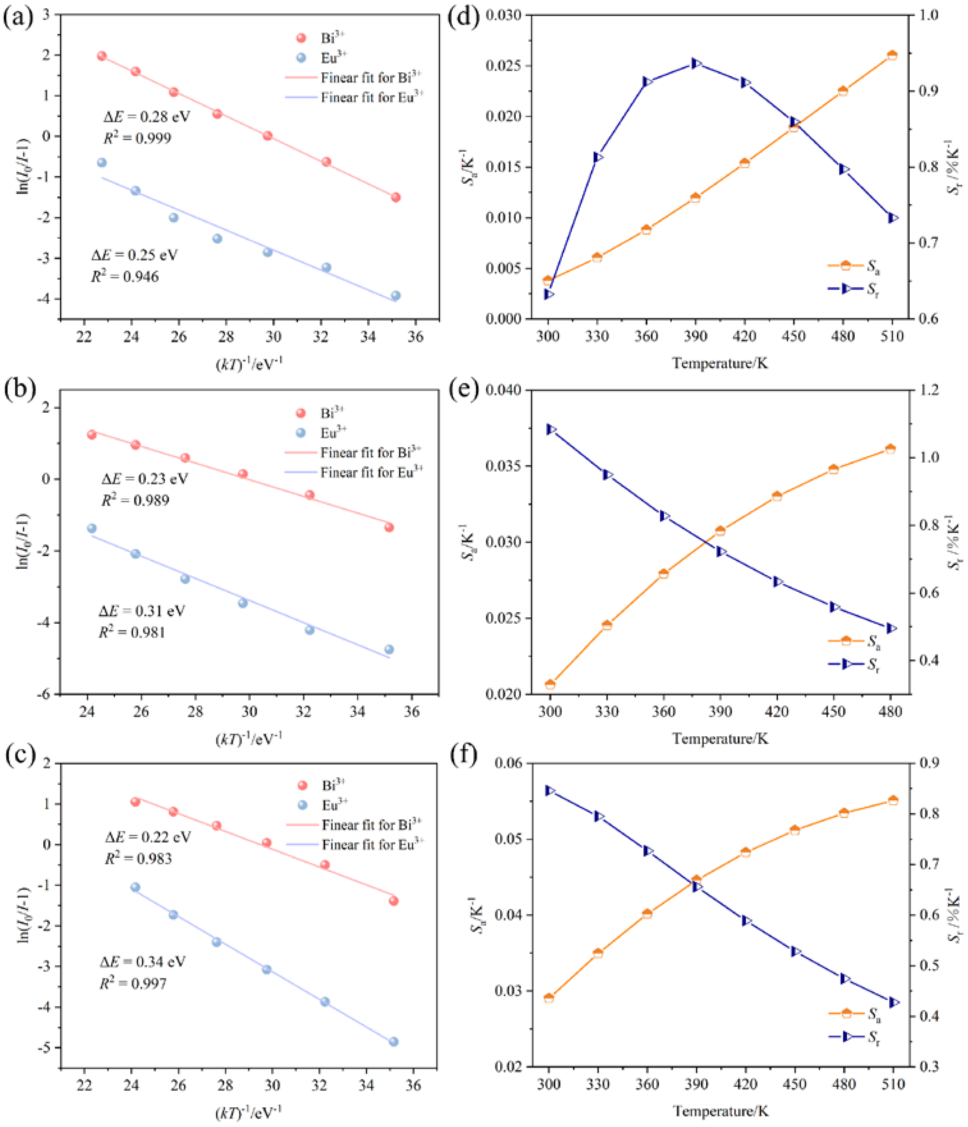

Fig. S4. Linear fitting of ln(I_0/I-1) versus (kT)$^{-1}$ (a-c) and *The* S_a *and* S_r *versus* absolute temperature (d-f) of CTO:0.04Bi^{3+}/$y$$Eu^{3+}$ (y = 0.02, 0.04, 0.06).

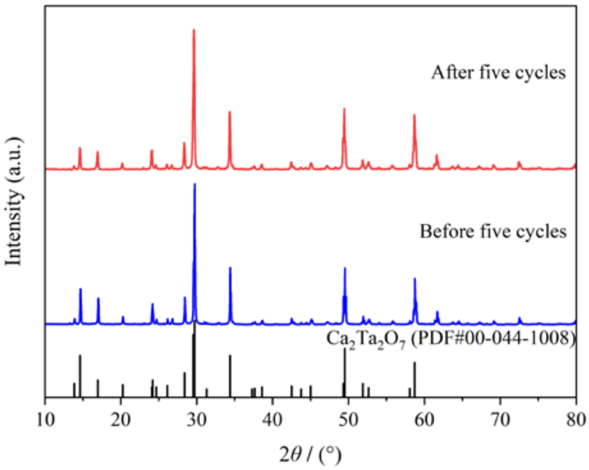

Fig. S5. XRD patterns of CTO:0.04Bi^{3+}/0.01Eu^{3+} before and after five cycles.

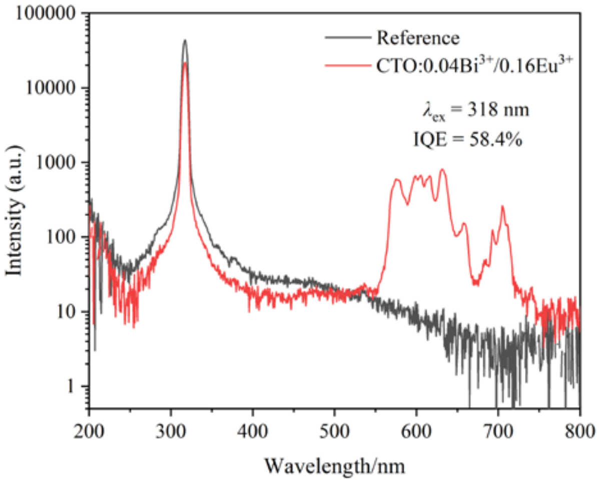

Fig. S6. PLE line of $BaSO_4$ and PL spectrum of CTO:$0.04Bi^{3+}/0.16Eu^{3+}$.

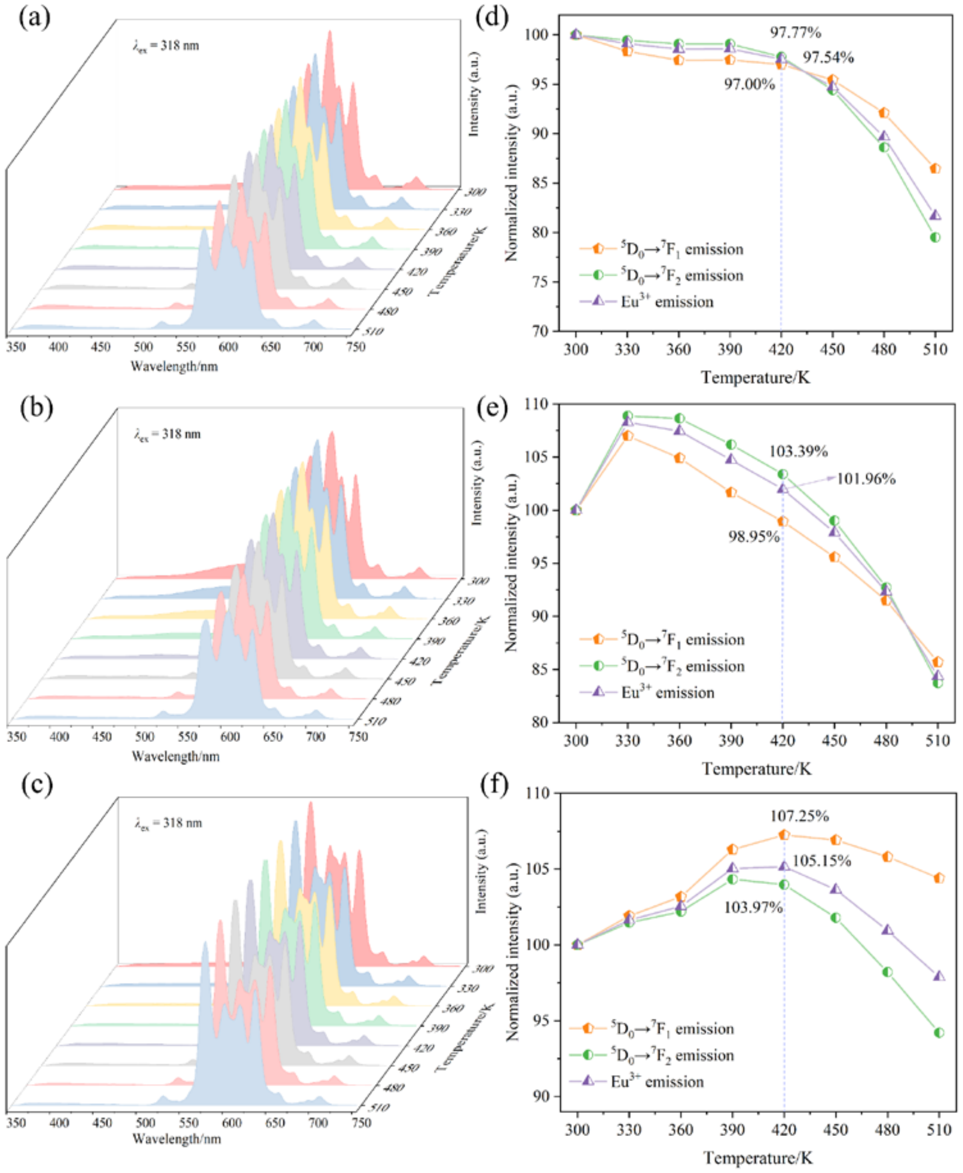

Fig. S7. Temperature-dependent PL spectra (a-c) and normalized PL intensity based on various temperatures (d-f) of CTO:$0.04Bi^{3+}/yEu^{3+}$ (y = 0.08, 0.12, 0.20).

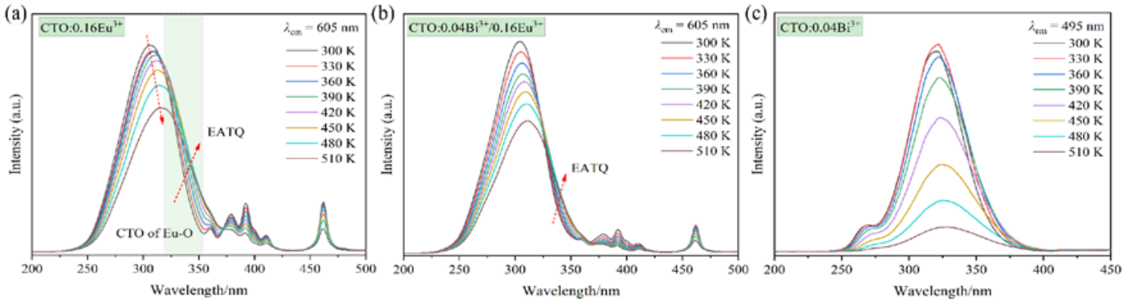

Fig. S8. Temperature-dependent PLE spectra of CTO:0.16Eu^{3+} (a), CTO:0.04Bi^{3+}/0.16Eu^{3+} (b), and CTO:0.04Bi^{3+}(c).

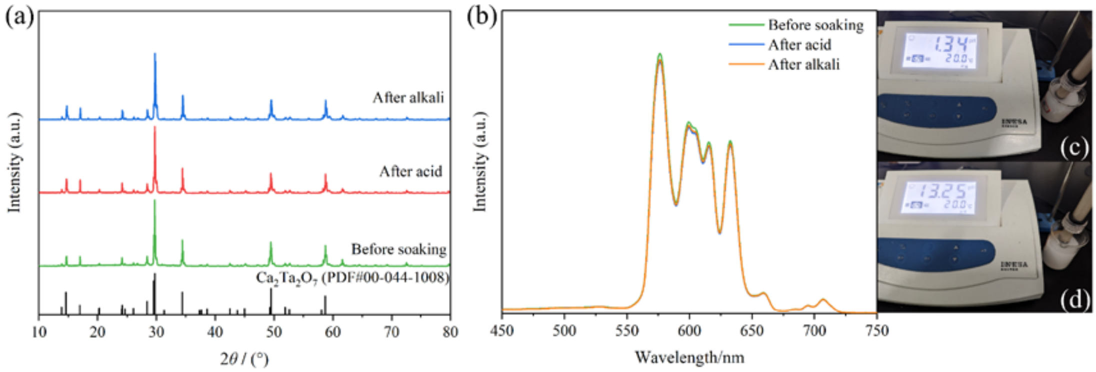

Fig. S9. XRD patterns (a) and PL spectra (b) of CTO:0.04Bi^{3+}/0.16Eu^{3+} before and after different immersion treatment; Photographs of CTO:0.04Bi^{3+}/0.16Eu^{3+} immersing in different pH solutions (c, d).

Reference

[1] Hafner J. *Ab-initio* simulations of materials using VASP: Density-functional theory and beyond. *J. Comput. Chem.* 2008;29:2044.

[2] Blöchl PE. Projector augmented-wave method. *Phys. Rev. B* 1994;50:17953.

[3] Perdew JP, Burke K, Ernzerhof M. Generalized Gradient Approximation Made Simple. *Phys. Rev. Lett.* 1996;77:3865.

[4] Grimme S. Semiempirical GGA-type density functional constructed with a long-range dispersion correction. *J. Comput. Chem.* 2006;27:1787.

[5] Monkhorst HJ, Pack JD. Special points for Brillouin-zone integrations. *Phys. Rev. B* 1976;13:5188.

[6] Wang V, Xu N, Liu JC, Tang G, Geng WT. VASPKIT: A User-Friendly Interface Facilitating High-Throughput Computing and Analysis Using VASP Code. *Comput. Phys. Commun.* 2021;267:108033.
